# Non-invasive magnetic resonance-guided high intensity focused ultrasound ablation of a vascular malformation in the lower extremity: a case report

**DOI:** 10.1186/s40349-015-0042-7

**Published:** 2015-12-30

**Authors:** Johanna M. M. van Breugel, Robbert J. Nijenhuis, Mario G. Ries, Raechel J. Toorop, Evert-Jan P. A. Vonken, Joost W. Wijlemans, Maurice A. A. J. van den Bosch

**Affiliations:** Department of Radiology, University Medical Center Utrecht, Utrecht, The Netherlands; Department of Vascular Surgery, University Medical Center Utrecht, Utrecht, The Netherlands

**Keywords:** MR-HIFU, Arteriovenous malformation, Vascular malformation, Non-invasive treatment, Clinical patient

## Abstract

**Introduction:**

Therapy of choice for symptomatic vascular malformations consists of surgery, sclerotherapy, or embolization. However, these techniques are invasive with possible complications and require hospitalization. We present a novel non-invasive technique, i.e., magnetic resonance-guided high-intensity focused ultrasound (MR-HIFU) ablation, for the treatment of a vascular malformation in a patient. This technique applies high-intensity sound waves transcutaneously to the body and is fully non-invasive. MRI guidance is the novel aspect of HIFU treatments and is used for exquisite delineation and localization of the lesion and accurate real-time temperature monitoring during tissue ablation. MR-HIFU is a well-established treatment option for uterine fibroids and is currently being investigated for, e.g., bone tumors, breast cancer, prostate cancer, and liver cancer. MR-HIFU of vascular malformations has not been a topic of research yet.

**Case description:**

Volumetric MR-HIFU ablation of a vascular malformation in the lower extremity of an 18-year-old male patient was performed. Temperatures of 62–80 °C were reached in the target lesion with sonications of 4 × 4 × 8 mm using powers of 200 W for <20 s. At 1-month follow-up, the patient reported qualitatively sustained reduction of pain and normal motor function. Three-month follow-up imaging indicated successful nidus destruction, which resulted in reduction of >30 % of the tumor volume. After 13 months, pain score was reduced to <2 after extreme exertion for several hours and to 0 for daily activities.

**Discussion and evaluation:**

Radiofrequency ablation and cryoablation are minimally invasive techniques that have been tried on low-flow vascular malformations with inconsistent results. Furthermore, both techniques require probe insertion, which is associated with risks of wound infection and hospitalization. Since MR-HIFU is truly non-invasive, these risks are negligible.

**Conclusions:**

In conclusion, we reported a successful non-invasive treatment of a vascular malformation with MR-HIFU in a clinical patient including long-term follow-up data for the first time. The patient reported qualitatively sustained pain reduction up to 13 months post treatment.

## Background

Vascular malformations (VMs) and tumors comprise a wide, heterogeneous spectrum of lesions and have been categorized by several classifications systems, including Hamburg’s classification [1]. Presenting symptoms range widely from no clinical signs to life-threatening congestive heart failure in both adults and children. The prevalence of VMs in the general population is estimated to be 1.5 % [2]. VMs occur in the head and neck (40 %), extremities (40 %), and trunk (20 %). Of all malformations located outside the central nervous system, 90 % is low flow (venous malformation) and 10 % is high flow (e.g., arteriovenous malformations) [3].

Therapy of choice for vascular malformations largely depends on the flow speed classification of the lesion. Image-guided sclerotherapy and surgical resection are recognized as standard therapeutic options for low-flow, venous malformations [4]. Therapeutic alternatives are being developed for recurrence after sclerotherapy or for vascular anomalies with mainly solid components such as fibro-adipose vascular anomalies, which could be inaccessible by sclerotherapy [5]. Sclerotherapy is not effective for high flow malformations as the injected agents are rapidly washed away from the endothelial lining of the nidus [3]. The cornerstone treatment for high flow lesions is transarterial embolization with occasional subsequent surgical resection [6, 7].

Lee et al. [8] state that a multidisciplinary team approach should be utilized to combine surgical and non-surgical interventions for optimum care. Current treatments, such as surgery and sclerotherapy, are associated with significant risk of morbidity and complications. Ligation of feeding arteries or coil embolization may result in proliferation of the nidus and would prevent future endovascular access. Inoperable lesions may be treated with embolo-sclerotherapy agents of which ethanol shows the best results and minimum recurrence. However, this technique requires extensive training and experience to minimize complications and morbidity. Surgical resection gives a chance of optimal control for operable lesions. Preoperative sclerotherapy or embolization may reduce the morbidity caused by, e.g., operative bleeding. This combined approach provides a potential for a curative result but is associated with risks of complications and morbidity and a high patient burden due to the invasive character of both procedures.

Recently, a growing interest can be observed for a novel technique to non-invasively treat tumors that were otherwise treated with surgical excision: magnetic resonance-guided high-intensity focused ultrasound (MR-HIFU). MR-HIFU is a well-investigated treatment option for uterine fibroids and is currently being investigated for, e.g., bone tumors [9], breast cancer [10], and liver cancer [11].

Ghanouni et al. [12] from Stanford University presented results of MR-HIFU ablation of slow-flow vascular malformations obtained in four patients during the 15th International Symposium of Therapeutic Ultrasound 2015 in Utrecht, The Netherlands. The average ratio of the non-perfused volume to the total tumor volume was 2.6 (range 0.75–7.4). No follow-up data were presented. Long-term follow-up data are of paramount importance with respect to VMs as they tend to regrow upon partial removal and symptoms may deteriorate.

We report for the first time MR-HIFU ablation of a venous malformation in the lower extremity in a clinical patient including follow-up of over 1 year post treatment.

## Case presentation

### Case

An 18-year-old male with no significant past medical history presented with pain (pain score of 8) in the medial side of his left lower limb. The symptoms had been present for over 10 years and were exacerbated by walking or running to the extent of pain preventing from any form of movement and/or exercise. No trauma had preceded the complaints. Upon physical examination pressure-induced pain of the medial side of the lower leg of the musculus gastrocnemius was observed. Minor swelling at the same location was palpable.

**Fig. 1:**
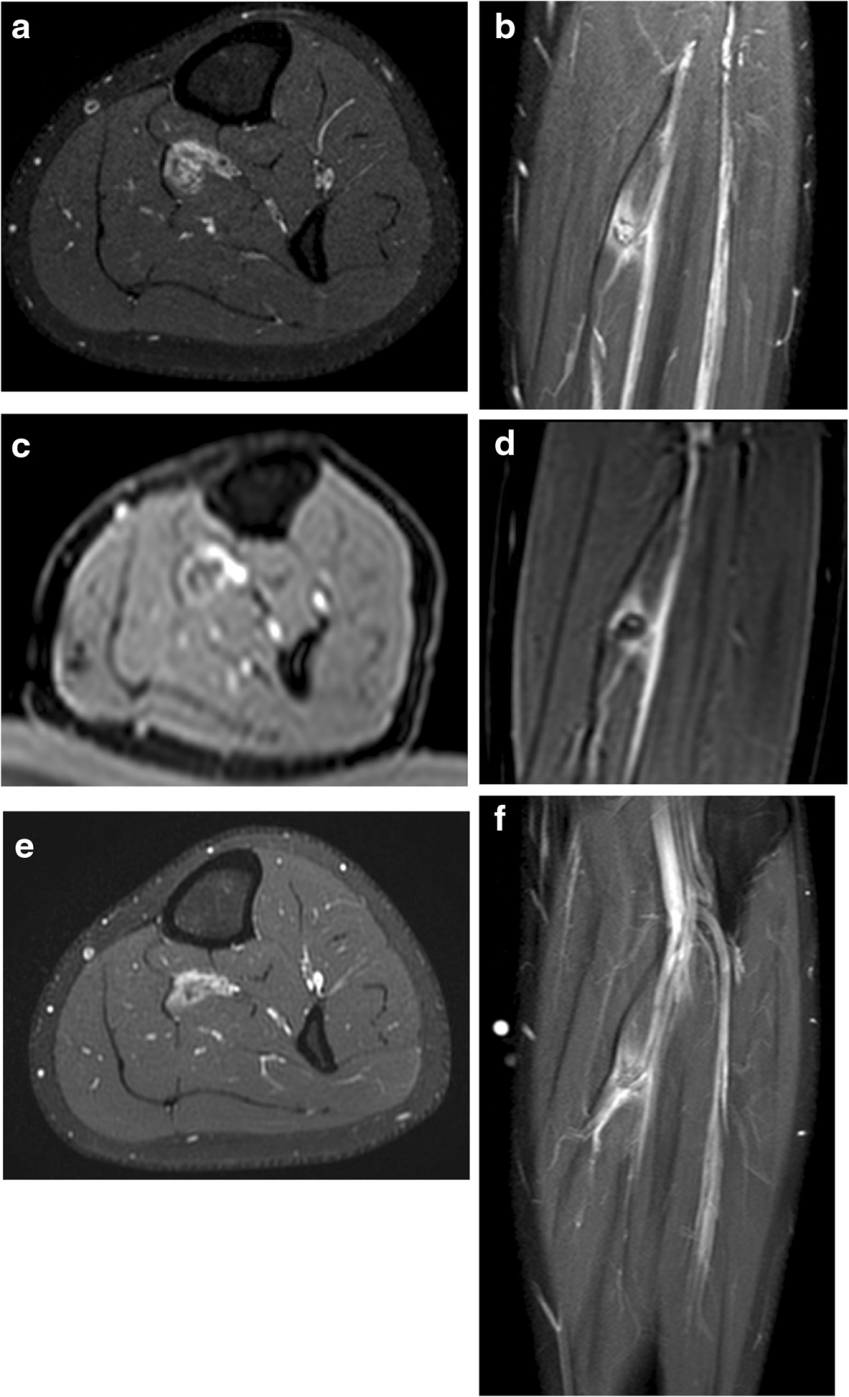
Contrast enhanced scans (axial view in **a**, **c**, **e** and sagittal view in **b**, **d**, **f**) several months prior to treatment (**a**, **b**), directly after treatment showing a non-perfused volume (**c**, **d**), and at 3 months follow-up showing a reduction in size of the malformation (**e**, **f**)

**Fig. 2:**
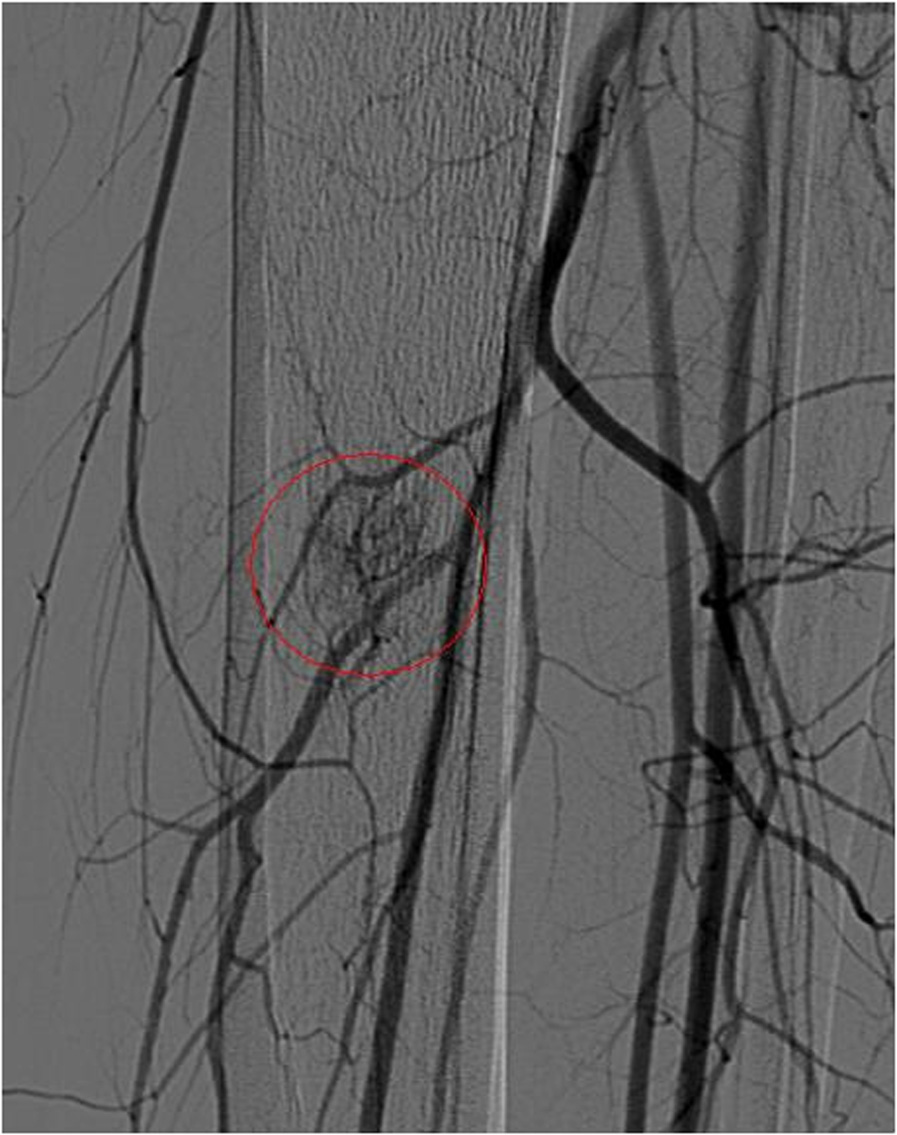
Angiogram of the left lower limb. The *red line* indicates the location of the vascular malformation

Several months prior to treatment, ultrasound examination was performed, as well as screening MR scans including contrast enhanced scans (T1 TSE spectral presaturation with inversion recovery (SPIR), Fig. 1a, b), and a digital subtraction angiogram (MRA) (Fig. 2). The lesion had a volume of 1.9 mL. Contrast injection showed inhomogeneous enhancement. An additional X-ray did not show any calcifications within the lesion. The swelling was characterized as a soft tissue mass of the musculus tibialis posterior with a feeding vessel branching from the arteria tibialis posterior and shunting to the vena tibialis posterior. No connection with the nervus tibialis posterior was observed. The malformation was characterized as an venous malformation in the lower limb (Hamburg’s classification). Biopsy of the lesion confirmed this diagnosis. The position of the vascular malformation with respect to nerves and main vessels (>2 mm) and the skin (>2 cm) was decided to be suitable for non-invasive treatment with MR-HIFU. The patient did not want to undergo surgery or an embolization procedure and signed informed consent for the MR-HIFU treatment described in this report.

### Treatment

**Fig. 3:**
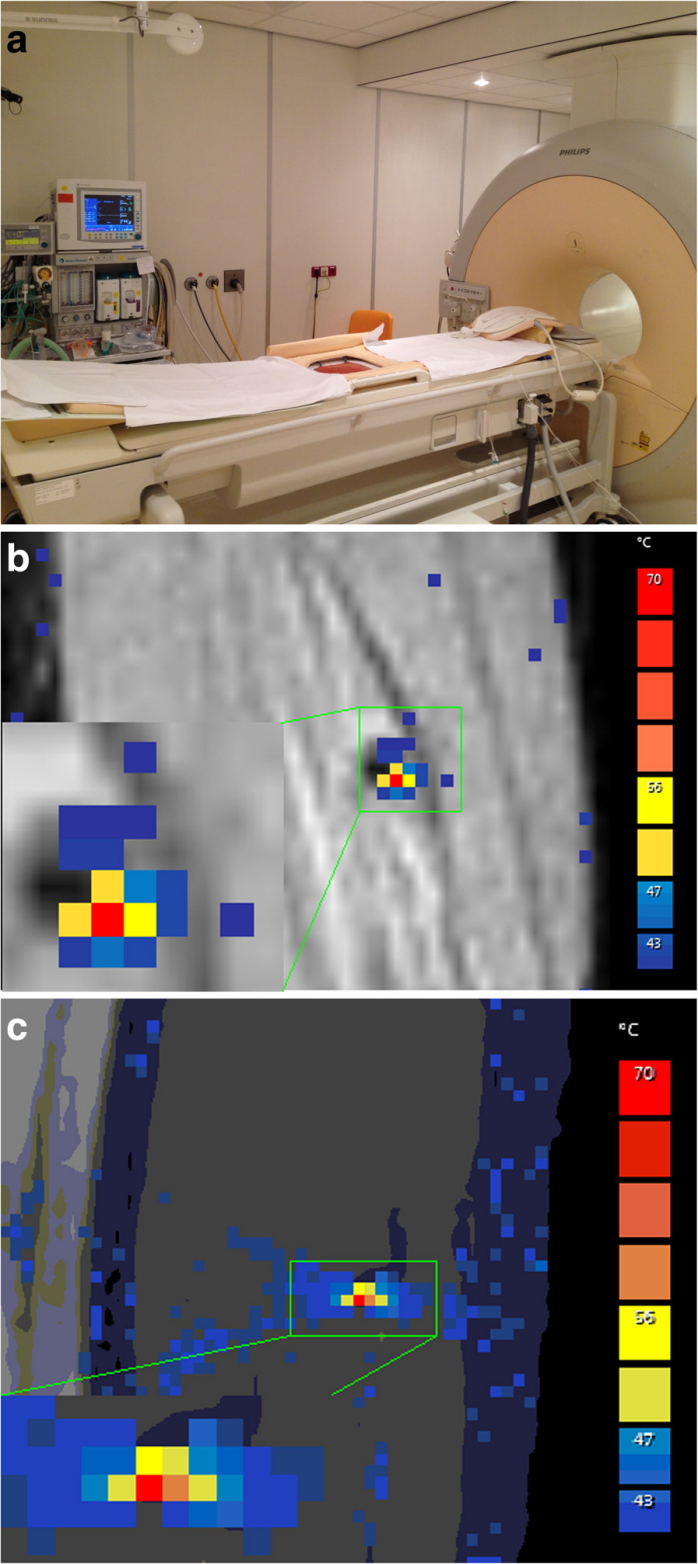
Set-up of the HIFU including the device for active skin cooling, which is integrated in the MR scanner (**a**). Temperature measurement during HIFU treatment. **b** Coronal and (**c**) sagittal views

At the patient’s request, the procedure was performed under full anesthesia. It should be noted, however, that this is not the preferred method of approach under normal circumstances for this type of malformation as this procedure could also be performed under conscious sedation. The patient was positioned in supine position on a 1.5 T MR-HIFU Sonalleve system (Philips Healthcare, Vantaa, Finland). An actively cooled water cushion provided skin cooling and enhanced acoustic coupling (Fig. 3a). First, a T2-weighted planning scan was performed for treatment planning. Therapeutic ablation consisted of five point ablations (4 × 4 × 8 mm, 200 W, duration 8.3–19.5 s), which were planned to cover as much volume of the vascular malformation as possible while keeping a safety margin (2 mm) from the adjacent nerve and vessels. During ablation, MR thermometry provided near real-time temperature mapping of the target area and adjacent tissues. This allowed the physician to observe the heating in and outside the target area (Fig. 3). Temperatures of 62–81 °C were reached during the ablation procedure.

At the end of the procedure, a contrast enhanced MR scan (0.1 mmol/kg body weight Gadovist®, Bayer Pharma, Berlin, Germany) was performed for treatment evaluation (MPR THRIVE for the transversal plane and T1 THRIVE SENSE for the sagittal plane). When comparing the pre- and post-treatment contrast-enhanced scans, the targeted region within the vascular malformation showed no enhancement after treatment (Fig. 1c, d).

### Follow-up

Directly after treatment, the patient experienced complete relief of pain symptoms, and physical examination showed no signs of neurological deficits. At 1-month follow-up, the patient reported qualitatively sustained reduction of pain (a score of 0 for daily activities and <2 for exertion) and normal motor function and sensation during a consultation with a vascular surgeon. At 3-month follow-up, a contrast-enhanced scan (T1 SPIR) was performed (Fig. 1e, f), which showed a decrease in volume of the lesion of >30 % (rest volume of 1.3 mL, software Osirix, Pixmeo, Switzerland). The malformation showed large non-perfused parts within the lesion (not excluded from the volume measurement). The part of the vascular malformation that was targeted with HIFU showed a large decrease in size, whilst the part adjacent to the nerves and main vessels, which was not targeted, was still intact (Fig. 1e, f). Furthermore, the patient reported qualitatively sustained pain reduction after 3 months and after 13 months with a visual analog scale (VAS) score of 2 only after several hours of exertion and no pain in rest or after any daily activity.

### Discussion and evaluation

In this case report, we demonstrated the feasibility and efficacy of the non-invasive MR-HIFU ablation of a venous malformation in a patient for the first time with follow-up data of up to 1 year. Post-treatment MR imaging showed a clear non-perfused center of the vascular malformation, indicating successful nidus destruction, which resulted in a volume reduction of at least 30 % after 3 months. Clinical follow-up showed no adverse effects, and the patient reported a pain reduction compared to baseline for up to 13 months post treatment.

Ghanouni et al. [12] presented results of MR-HIFU treatments of four patients with vascular malformations. The vascular malformations were located in the thigh or calf at an average focal depth of 14.9 cm. An ExAblate MR-HIFU system was used, which creates point ablations contrary to the volumetric approach used in this case study. The average treatment time was 2 h 35 min compared to 45 min for our patient treatment, and the average number of sonications per patient was 52 compared to 5 sonications in our case. Patients were treated while receiving general anesthesia and/or after peripheral nerve blockade. The average power used was 187 W (up to 253 W) and the average sonication duration was 9.7 s (up to 13.2 s), which is lower and shorter, respectively, than in our study due to the point ablation technique. The average of temperatures achieved was 49 °C. The maximum temperature was 54 °C. The golden standard for tissue ablation is a temperature of at least 56 °C [13], and this is what we aimed for during our ablations to ensure cell death and/or vessel occlusion within the venous malformation. Ghanouni et al. did not show follow-up data including volume reductions, non-perfused volumes, etc. However, this is of paramount importance as VMs may regrow upon incomplete resection thereby possibly deteriorating symptoms. Several explanations have been described in literature for VM regrowth [14–16]. Pellettieri et al [15] introduced the concept of hidden compartments: unfilled compartments that may exist within a VM but are not seen on angiograms—the modality that is the accepted standard in order to conclude that full resection has been accomplished. These compartments can have separate feeders and drainage and may get filled after hemodynamic changes due to, e.g., embolization or MR-HIFU. This might account for recurrence or growth of the (A)VM. It was observed in five cases that unorganized abnormal vasculature can recruit new, small and low current feeding vessels from a distant location that finally form a reorganized (A)VM at the original site, which presents as regrowth of the (A)VM [17, 18].

Another possible explanation is the action of multiple cell-derived and extracellular factors and in particular vasogenic factors [19–23]. Altered levels of angiopoietin-2 and vascular endothelial growth factor have been detected in the venous drainage system of AVMs [24]. Stenosis leading to venous dysfunction may also play a role in (A)VM growth probably due to hypoxia/local ischemia [24]. Ironically, these effects can be induced by current and newly researched treatments, such as embolization and MR-HIFU.

Other novel techniques used to treat VMs are radiofrequency ablation (RFA), cryoablation, and laser ablation, all minimally invasive treatment options. RFA was performed in three patients with low-flow soft-tissue vascular malformations and was clinically successful in two patients [25]. Furthermore, Childs et al. [5] and Berber et al. [26] described the successful treatment of venous malformations in one patient each with RFA. A study by Gao et al. [27] on 16 patients with venous malformations showed that RFA treatment resulted in complete resolution of the vascular malformation (>90 %) in only two patients. Less or no success was achieved in 14 patients. No serious complications were observed in any of these cases.

Cryoablation has been described to successfully treat a pectoral venous malformation [28]. Furthermore, a study on four patients with venous malformations in the right calf, right limb, left pectoral region, and lumbar region has shown promising results, i.e., a mean volume decrease of 95 % of the lesions after 6 months [29]. Another study performed cryoablation or laser ablation under US/MRI/CT guidance on eight patients with nine slow-flow vascular malformations. Two minor complications were reported: a small intramuscular hematoma and numbness of the dorsal aspect of the first toe. No intervention was needed in both cases. Average follow-up time was 19.8 months, and all patients reported (for one patient a retreatment was required) symptomatic relief [30].

Even though RFA, laser, and cryoablation are minimally invasive techniques, all three need insertion of a probe into the lesion [28, 29]. MR-HIFU, on the contrary, is completely non-invasive, eliminating certain risks associated with invasive procedures such as wound infections. The treatment can be performed under general anesthesia or deep sedation. The advantage of HIFU over minimally invasive techniques such as RFA and cryoablation is the real-time guidance and feedback with MRI during the procedure, which provides accurate lesion delineation and temperature feedback resulting in more accurate heating.

A limitation of MR-HIFU is that the lesion may not be accessible by the ultrasound beam, for example, when bone or nerves are located within the treatment beam. This can largely be overcome by positioning the patient such that no critical structures are in the beam path. Lesions in the thorax and the head can be a contra-indication as air causes scattering of ultrasound. Most locations of vascular malformations are however in the head/neck (40 %) and extremities (40 %) [31].

A known challenge in clinical practice is successful ablation of high perfused lesions due to the so-called heat-sink effect [32–34]. Cooling by blood flow limits the temperature rise in the focal point and thereby may lead to insufficient cell damage and/or vessel occlusion. The heat-sink effect was not very prominent in our case, and the temperature rise in the focal point was not limited. Hynynen et al. [35] and Voogt et al. [36] suggested that the mechanism for vessel closure is probably first a mechanical/thermal stimulus to the vessel that causes transient constriction. This eliminates the heat-sink effect by blood and thus secondly, the vessel wall can be thermally coagulated by consequent sonications.

## Conclusions

In conclusion, we have reported a successful treatment of a vascular malformation with MR-HIFU, a new, completely non-invasive technique currently used in clinical practice for tumor ablations. MR-HIFU allows accurate delineation of the vascular malformation and real-time temperature feedback during treatment. This allows accurate targeting of the lesion and sparing of the surrounding healthy structures. We believe this is a promising technique for future treatments of vascular malformations.

## Abbreviations

AVM: arteriovenous malformation
MRA: magnetic resonance angiogram
MR-HIFU: magnetic resonance-guided high-intensity focused ultrasound
RFA: radiofrequency ablation
SPIR: spectral presaturation with inversion recovery
TSE: turbo spin echo
VM: vascular malformation
